# Facilitators and barriers faced by community organizations supporting older adults during the COVID-19 pandemic

**DOI:** 10.1186/s12877-025-05816-w

**Published:** 2025-03-28

**Authors:** Kristen R. Haase, Ailsa C. Sirois, Dmitri Detwyler, Bahareh Kardeh, Shelley Peacock, Theodore D. Cosco, Marjan Kamali, Megan E. O’Connell

**Affiliations:** 1 School of Nursing, Faculty of Applied Science, University of British Columbia, T201-2211 Wesbrook Mall, Vancouver, BC V6T 2B5 Canada; 2 Cancer Control, BC Cancer Research Institute, Vancouver, Canada; 3 Department of Applied Linguistics and ESL, Georgia State University, Atlanta, USA; 4 Faculty of Medicine, Graduate Program in Rehabilitation Sciences, University of British Columbia, Vancouver, Canada; 5 College of Nursing, University of Saskatchewan, Saskatoon, Canada; 6 School of Gerontology Research Centre, Department of Gerontology, Simon Fraser University, Vancouver, Canada; 7 Oxford Institute of Population Ageing, University of Oxford, Oxford, UK; 8 Department of Psychology, University of Saskatchewan, Saskatoon, SK Canada

**Keywords:** Aging, COVID-19, Community organizations, Information technology

## Abstract

**Background:**

During the COVID-19 pandemic, olderadult-focused community organizations played an essential role in supporting the wellbeing of older adults. Supporting older adults during this time required extensive modifications to existing programming but their adaptations during the COVID-19 pandemic are not well documented. The purpose of this study was to understand how older adult-focused community organizations adopted virtual delivery formats during the COVID-19 pandemic and their perspectives of the barriers and facilitators for organizations and older adults.

**Methods:**

To understand the changes that were made, we conducted a qualitative environmental scan of community-based services across British Columbia. Online searches were complemented by snowball sampling and key informant interviews. We identified 90 older adult-serving community organizations and interviewed 26. We used reflexive thematic analysis to understand the main strategies.

**Results:**

These community organizations described barriers related to older adults’ wellbeing, information technology proficiency, and personal/organizational losses related to changes in program structure. Facilitators for virtual activities and events included inter- and intra-organizational collaboration, intrinsic qualities of program design, physical resources to supporting virtual programming, and availability of technological resources. Organizations described meeting the challenge by increasing the ‘depth’ and ‘breadth’ of their reach.

**Conclusion:**

Older adult-focused community organizations recognized the critical role they played for older adults and adapted their resources to meet those needs. Informational technology was quickly and effectively leveraged to promote social interaction for older adults when physical distancing was required during the COVID-19 pandemic. Barriers related to cost, time, and ultimately older adults’ interest in a virtual delivery format were critical limitations.

**Graphical Abstract:**

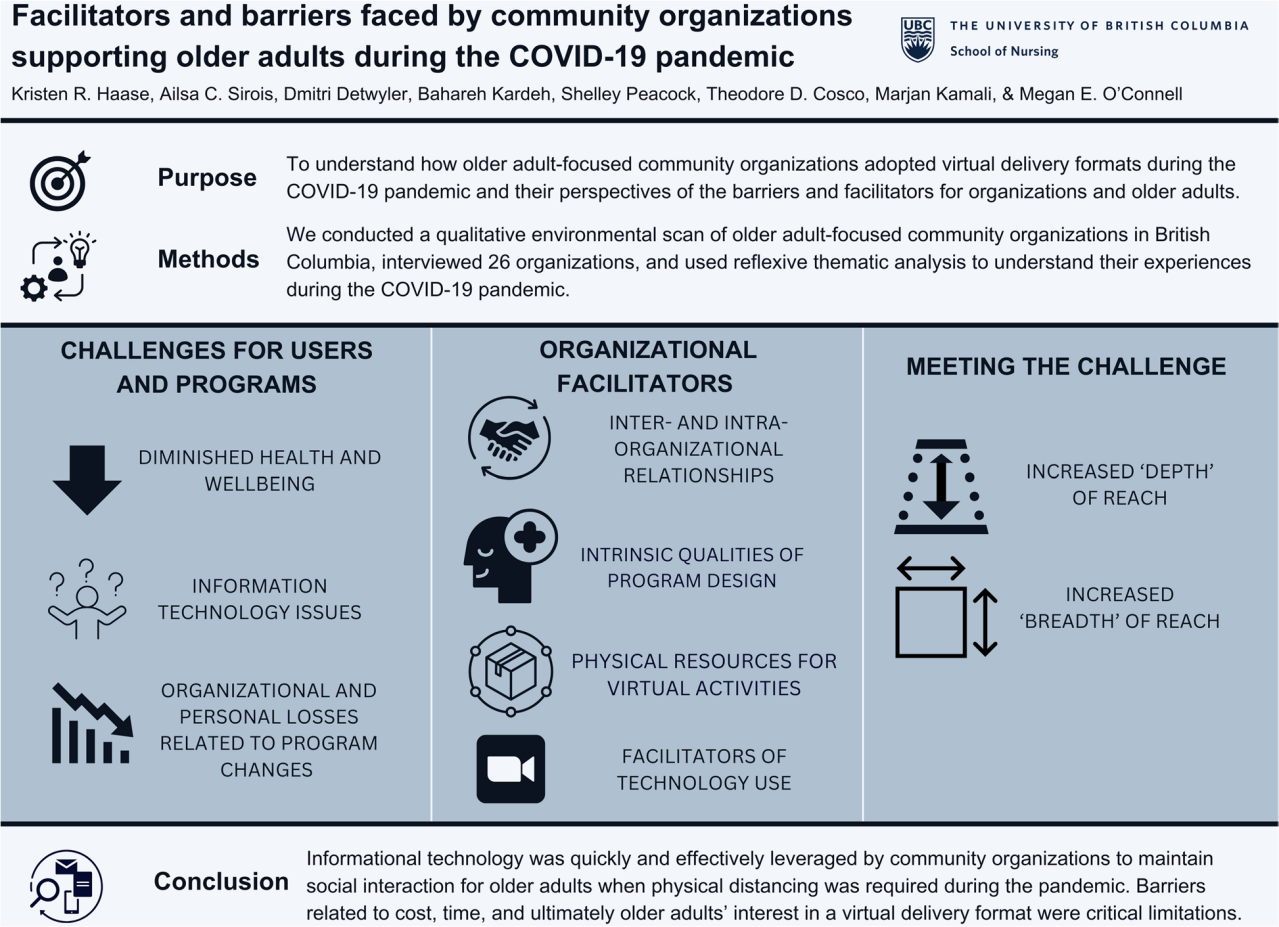

**Supplementary Information:**

The online version contains supplementary material available at 10.1186/s12877-025-05816-w.

## Background

Over the course of the COVID-19 pandemic, public health restrictions regarding physical distancing were implemented globally to protect high-risk populations including older adults. These measures were intended to protect older adults but inevitably reduced in-person interactions and community participation, leading to adverse consequences such as social isolation and loneliness among some older adults [1–3]. In Canada, health centres and essential services mostly remained open during the pandemic to provide necessary services either in person or virtually via both internet and telephone supported services. Many social and community organizations, including senior centers (who provide critical non-medical services) were mandated to close in-person services [4]. It could be argued, however, these resources should have been characterized as essential, given that lack of social support can lead to physical decline and poor health [5, 6].

For older adults, community organizations play a critical role in wellbeing by providing services that promote physical health, including chronic illness management [7, 8] and preventive care [9]. These organizations also play a key role in promoting leisure activities, helping older adults maintain a structured routine, and fostering a sense of community [10–12]. Engaging in such activities to maintain physical and cognitive functions is a component of ageing well [13–15]. Thus, community organizations may improve older adults’ quality of life as they allow older adults to develop social networks and combat isolation and loneliness [16–18].

A national survey from the Canadian Mental Health Association notes that many mental health focused community organizations faced challenges delivering services during the COVID-19 pandemic and felt that older adults were particularly disadvantaged in the move to virtual services [19]. Information technology (IT) use during the pandemic increased among older adults and remains on an upward trajectory [3, 20]. Evidence related to the acceptance and perceived usefulness of IT among older adults remains mixed [3, 21]. In a small phenomenological study, older adults who were able to access virtual community centre programs reported that IT was a valuable tool for participating in programs such as book clubs and support groups [2]. Wolman and colleagues reported that virtual offerings were particularly well-received among those with mobility issues but overall, older adults missed in-person programs and ranked online options as ‘second best’ [2]. Unfortunately, the perspectives of older adults who were averse to, or unable to engage in virtual programs were not included in this study by Wolman and colleagues (a notable limitation) [2].

To understand the most efficient and meaningful way to support the health and wellbeing of older adults during the pandemic, we sought to document the efforts that took place amongst community organizations operating in different contexts within the Canadian province of British Columbia (B.C.), to meet the needs of older adults via virtual delivery methods. This paper contributes to the evidence regarding older adult-focused community organizations’ perspectives of utilizing virtual programming to support older adults during this period [4, 22].

### Purpose

The purpose of this study was to determine the extent to which older adult-focused community organizations moved to online or virtual delivery formats and to document these organizations’ perspectives of the nuances of the barriers and facilitators to moving to a virtual format for both the organizations and older adults. The research questions were: 1) To what extent did older adult-focused community organizations in B.C. move to online or virtual delivery formats to support the social needs of older adults during the COVID-19 pandemic? 2) What are the perceptions of older adult-focused community organizations on the barriers and facilitators to moving to online or virtual delivery formats during the COVID-19 pandemic?

## Methods

We conducted an environmental scan of community organizations for older adults in one Canadian province. Environmental scanning is a method designed to gather information about an organizational ecosystem related to opportunities and threats, to inform future organizational strategies [23, 24]. It is a suitable method to understand the strengths, weaknesses, and opportunities for improvement within a specific context [25]. The environmental scan method aligned with our study aims and was feasible within the constraints of the study (i.e. during the pandemic with limited resources and the need to generate information using distance-based methods and within short timeframes to be able to quickly intervene) allowing us to understand the full landscape of services offered by older adult-focused community organizations. This study was reviewed and approved by the University of British Columbia Harmonized Research Ethics Board (H20-03120).

### Setting and sample

The study was conducted in the Canadian province of B.C., comprising five geographically and demographically unique regions. We aimed to recruit representatives from a sample of older adult-focused community organizations from all five regions which included urban, rural, remote, and northern locations. We recruited participants until we noted repetition in themes related to our research question. While sampling is discussed in environmental scan methods, there is limited guidance on sample size. We adopted principles of information power [26] and theme repetition [27] which we revisited throughout the data collection process. After 22 interviews, there was consensus on the themes and repetition in the experiences shared by the organizations, and we felt they sufficiently answered the research questions. We sought to conduct four additional interviews to ensure that additional nuances were not missed.

### Data collection

We collected data via internet searches and interviews with key informants. In line with environmental scanning approaches, we used multiple strategies to identify community organizations and key informants which included internet searching and snowball sampling [25]. This process started with searches on Google and Facebook for ‘senior organization’ (senior OR seniors OR older adults) AND ‘British Columbia’. For each result, we collected publicly available information about the organization’s location, purpose, and prospective key informant email addresses and phone numbers. This information was tracked in an Excel spreadsheet and organized by the province’s five public health regions, to ensure geographic coverage (some organizations had a province-wide reach, which was also noted). We expanded the list of organizations by searching a publicly available online directory of older adult-focused organizations called BC211 [28] and snowball sampling with organizations that agreed to participate. We searched the BC211 service by using the selections: older adults and social/recreation, and contacted all organizations who had listed contact information and were operating during the pandemic.

We generated a list of 90 organizations, of which 28 were found through web and social media searches, 34 were from the BC211 database, and 26 were identified from snowball sampling. We also contacted one organization for which a representative was known to the first author and another which was profiled in national news coverage for their virtual programming for older adults. We adopted a layered approach to searching, where new results from each additional strategy (web and social media, database, snowball sampling) were added to the spreadsheet. For each organization on the list, we sent a recruitment email to the identified contact person inviting them to participate in the study and followed up with a phone call if no response was forthcoming. We contacted a total of 90 organizations, of which 26 responded and agreed to participate. When a contacted person agreed to participate, we obtained their consent electronically and an author (DD) conducted a telephone or Zoom (a video-conferencing software) interview lasting 45 min on average.

Interviews were conducted with an executive director or suitable surrogate (i.e. program manager). We used a semi-structured interview guide that was developed with members of our team who have experience working with older adult-focused community-based services (see supplement for interview sample questions). Interviews were conducted by an experienced qualitative researcher (DD) who scribed the interviews by recording comprehensive notes, and summarizing participant responses in a table format. There is potential for loss of nuance in scribing compared to recording and transcribing. Nonetheless, a 2019 study found that themes identified in qualitative research where scribing was used was highly-consistent with themes identified from traditional recording and transcribing methods [29]. Scribing – whether by a third person as described by Eaton [30] or the interviewer themself – may allow researchers to synthesize data and include salient contextual details in their notes [29, 30]. This method aligns with our study design as it promotes an iterative process; preliminary data analysis during the scribing process informed subsequent interviews [30]. To further promote accuracy, we took the additional step of sending the completed table of scribed notes to participants to be checked for corrections or additions. Participant revisions were typically minor clarifications or addition of details. Because the data for this analysis was the researcher’s paraphrase rather than the participants’ own words, we use only limited verbatim excerpts in presenting the results below.

### Analysis

After the notes were reviewed by participants, they were imported into the latest version of NVivo (released March 2020) for analysis. We undertook an initial cycle of attribute coding for region, user population (seniors vs. all ages), and primary purpose (social, educational, health, or mixed), based on each organization’s public web site and participant responses [31]. Subsequently, two researchers engaged in a reflexive thematic analysis [27]: two authors (DD, KH) engaged in a process of reading and assigning codes, organizing data into themes, and defining and refining themes and sub-themes. The PhD-prepared authors involved in data analysis both have experience conducting qualitative data analysis and experience working with, and volunteering within community organizations. Throughout the analytic process, we developed inductive themes based on the meanings provided by participants [32].

We addressed rigour following the tenets of rigour and trustworthiness described by Thorne [33]. Although rigour is not often explicitly addressed in environmental scanning approaches, we used principles of: (1) interpretive authority; (2) epistemological integrity; (3) analytic logic; and (4) representative credibility [33]. We did so by ensuring our purpose, approach, and methodological decisions were aligned; limiting claims to those which could be supported by the scribed interview data; checking our interpretations of interviews with each participant; and clearly describing the limitations of our study.

## Results

From December 2020 through June 2021, we interviewed 26 contacts from 90 identified community organizations (response rate of 29%). Twenty-five community organizations had moved at least some services to a virtual format. Table 1 summarizes the number and geographic distribution of organizations that were contacted, and the number of those who agreed to participate in the study.

**Table 1:** Number and geographic distribution of organizations

	Fraser	Interior	Northern	Vancouver Coastal	Vancouver Island	Province-Wide
No. of orgs. contacted	15	16	6	33	18	2
No. of orgs. interviewed	7	3	1	12	3	0
Percentage	46%	19%	17%	36%	17%	0%

Based on interview responses and publicly available website information, we profiled participating community organizations by their constituency (older adult-focused vs. all-ages with some older adult-oriented programming) and purpose (educational, social, health, or mixed) as presented in Table 2. In our classification, the constituency of older adult-focused community organizations welcomed family caregivers who could be but were not necessarily older adults.

**Table 2:** Constituency and purpose of organizations

	Social	Educational	Health	Mixed
Older adult-focused	6	7	1	9
All-ages with older adult-focused programs	1	0	0	2

We identified three themes (with subthemes) that describe the complexities of shifting existing programs to virtual delivery formats and developing new programs under pandemic conditions from the perspectives of community organizations: (1) *challenges for users and programs*; (2) *organizational facilitators*; and (3) *meeting the challenge*. Under each theme we iteratively coded several sub-themes, and this process led us to recognize that ‘barriers’ impacted individuals and organizations alike. For example, community organizations provided unique perspectives on how their users faced barriers accessing services, and how they experienced barriers in providing services. 'Facilitators’ included factors that made it easier for the organization to provide services. The themes and subthemes supported by participants exemplars are described below (see Table 3 for thematic map).

**Table 3:** Thematic map

Theme	Subtheme	Data excerpt
1. Challenges for users and programs	1.1 Diminished health and wellbeing	“[We’ve seen] substantial declines in physical health and mobility, also cognitive health especially if they have pre-existing cognitive issues; mental health issues including sadness, depression, intense anxiety.”
		“Lots of issues around social isolation and loneliness with a high proportion of older adults living alone. There’s been a general uptick in mental health and wellness challenges and housing is also an issue despite eviction moratorium, rent freezes.”
	1.2 Information technology issues	“Gaps include access to wifi for low-income seniors, it’s not considered a necessity even though they feel like it would be helpful and are missing out. Knowing about devices is another difficulty (tablet vs. iPad vs. phone).”
		“Many elderly folks don’t have a device.”
		“The single biggest difficulty is an entire cohort of usual registrants whom [we] haven’t been able to reach – people who may get the email newsletter but don’t have the tech ability to get started with Zoom, or their devices are outdated, or they’re really scared of what Zoom is.”
	1.3 Organizational and personal losses related to program changes	“One female member had tried one class but didn’t like it because the social interaction wasn’t there, and that is the big thing that is keeping other women away.”
		“80–90% of activities are run by seniors for seniors as part of [our] non-profit society. About 200 volunteers lost roles due to activity cancellations.”
2. Organizational facilitators	2.1 Inter- and intra-organizational relationships	“The organization is just starting to plan some events to begin in June and are currently looking for community partners. Going to start with an arts program. The RCMP will do a session on scams targeting seniors and how to protect themselves; interviews and conversations with local seniors; further along, they are hoping to do movement, chair yoga, mindfulness, things to do for health and wellness; maybe a cooking class; currently gathering these ideas and starting to partner with different groups.”
		“[We try] to be mindful about identifying as part of [the university], and thankful because even just handling the enrollment is an enormous task. For those Elder Colleges that don’t have the postsecondary connection, it’s been tougher…. [We have] a couple of [university] program assistants, and part of their job is to support the Elder College. They produce the calendar, work with instructors to do the scheduling, and handle all the administrative and course management work behind the scenes. They also offered their services to organize and lead the Zoom Cafes… for anybody who wants to practice (students and instructors). They can try things, do Q&A, and so on.”
	2.2 Intrinsic qualities of program design	“They [seniors] are becoming aware, especially for seniors, that engagement through regular mail (Canada post) and telephone are still an important and good way to keep folks connected to their community.”
		“Another program is the 1:1 support, with weekly telephone calls (and for a few, daily check-ins by neighbors). It’s done mostly by students and volunteers up to the age of 81. Some of the older adults prefer an older person to call them. Then this winter [we] did a new initiative: the intergenerational program. [We] tried to be flexible with desires and wants of seniors and match students with them based on career goals, etc. One older adult is learning Excel through Zoom… One person is doing it by WhatsApp, because she’s actually communicating in Mandarin. We have some multilingual connections (Mandarin, Romanian, etc.).”
	2.3 Physical resources for virtual activities	“They [organization staff] have just launched an art program with a facilitator. They bought supplies and dropped them off / did pickups to get things started.”
		“Access to devices and availability to wifi [has been a challenge but we] fundraised for 34 tablets and worked with Shaw to put free wifi in building hallways.”
	2.4 Facilitators of technology use	“Support with technology and skills offered from multiple directions: Professionals from local businesses, school-age youth, and members with relevant skills from former working life to get set up on Zoom and use computing devices.”
		“When [our] building was closed at the start of the pandemic, the lifeguard staff served as the tech staff. “Lifeguards to the Rescue.” People could call in and get 1-on-1 help on the phone and connect with one another to play their favourite games.”
3. Meeting the challenge	3.1 Increased ‘depth’ of reach	“Seniors are nervous about Zoom programs going away after the pandemic. Online activities and food programs have been really meaningful, and [organizations have] found new users of their services. Organizations just didn’t realize how many people needed help (related to surgery recovery, lack of family support, limited knowledge or access to services). The pandemic facilitated the creation of new services. We need to keep it going.”
		“On Mondays [we] have a telephone program called the Lighthouse Program that turned out to be a hybrid – combining telephone and video conferencing. It used to be in-person for seniors with mild cognitive impairment, dementia living at home. We’ve reached out to facilities with a few Japanese residents, and some were very responsive. A dedicated a staff member brings the Japanese residents to the lounge and set up the video conference. There are currently have two facilities where some residents participate. Facilities with a limited number of Japanese speakers might have no activities for those residents. Long term care facilities have a mandate to provide culturally based programming and food. Our organization is helping to fill in this gap in mainstream facilities.”
		“We have three people at a residential facility down the road doing courses at our Elder College. They haven’t been able to before the pandemic and shift to online classes.”
	3.2 Increased ‘breadth’ of reach	“They no longer have a firm geographical boundary, one limited by driving distance. Have students and even instructors from other provinces, from the US (e.g., an instructor teaching from his second home in California).”
		“We’ve had conversations with other Elder Colleges about the issue of poaching students, but it hasn’t really happened. Everybody is welcoming this expansion of geographical boundaries. We expect to see a further geographical expansion for our courses.”

### I. Challenges for Users and Programs

Community organizations described various challenges as they attempted to address the social support and interaction needs of older adults during the pandemic through virtual program delivery. The challenges encompassed three main areas: (1) health and wellbeing; (2) information technology issues; and (3) organizational and personal impacts of program changes.

#### Diminished health and wellbeing

Many participants reported that social isolation and loneliness were the most salient challenges reported by their program users. However, they perceived the personal health challenges of their collective users as significant. Participants shared that program users reported dementia, cognitive decline, visual impairment, deafness, psychological trauma not due to but exacerbated by the pandemic (e.g., feelings of confinement among individuals who had experienced trauma), and mental health challenges such as anxiety and depression. Participants described how older adults’ pre-existing challenges were compounded by the pandemic which motivated community organizations to find novel ways to maintain their services via virtual delivery means. Some respondents also noted that members encountered challenges involving access to necessities such as food or housing.

Respondents serving linguistically and culturally diverse populations also raised the importance of cultural health and wellbeing. Participants were concerned that program users who spoke first languages other than English were at greater risk for missing information about public health restrictions and vaccinations. Additionally, one participant emphasized that public health measures had harmful implications for Indigenous older adults whose strong connections to family, community, and traditional lands had been disrupted.

#### Information technology issues

Challenges related to older adults using IT to connect remotely with community organizations were frequently shared. Older adults’ difficulty learning to use unfamiliar IT devices and platforms was a top reported challenge by community organizations. Participants observed that these difficulties could be exacerbated by ageing-related cognitive decline, memory loss, or dementia. Almost as prominent was lack of access to IT devices or internet connections at home. Within this category, participants emphasized the importance of distinguishing between older adults who seldomly used IT, and those with more familiarity but lost access when public computers or Wi-Fi (e.g., at libraries or cafés) became unavailable due to the pandemic restrictions. Several community organizations struggled to provide IT support for users seeking to get online and use remote programs exacerbated this situation. Another IT challenge described by participants was older adults’ generalized distrust of technologies grounded in concerns about showing one’s home surroundings on video; being spied on through cameras and microphones; and providing one’s financial information in new ways (e.g., paying a registration fee online for an online course). Some participants reported accessibility challenges with common IT device and platform interfaces, for example telephone conferencing platforms that were unintuitive to use, or tablets that were physically difficult to manipulate with reduced dexterity after a stroke.

#### Organizational and personal losses related to program changes

Participants described various organizational and personal losses related to the suspension of organized, in-person activities. The most notable loss for program users was the loss of informal social interactions such as chatting before or after a gathering and the loss of an enjoyable routine. Given that many organized activities such as classes were led by volunteers who are themselves older adults, the suspension of gatherings also led to role loss for some, especially if they were unable or unwilling to make the transition to facilitating their activities online. Thus, these program changes had both program-level and personal implications.

### II. Organizational Facilitators

Despite facing many challenges, several organizational attributes and resources facilitated participants’ ability to continue supporting older adults: (1) inter- and intraorganizational relationships; (2) intrinsic qualities of program design; (3) physical affordance of virtual activities; and (4) technological facilitators.

#### Inter- and intra-organizational relationships

One of the most frequent and reportedly impactful facilitators for providing successful online activities was collaborations with other older adult-focused community organizations. Cooperation among organizations sharing a similar focus led to exchanging knowledge and best practices, and member referrals. New partnerships with other community organizations resulted in new virtual programming, including virtual tours of museums or art galleries; at-home exercise routines led by yoga studios; and reading clubs co-hosted by libraries. Another prominent form of cooperation was to rely on another, often larger, organization to provide IT training and support for members.

Several community organizations interviewed for this study were older adult-focused programs within a larger organization providing services to people of different ages. An example of this relationship was so-called ‘Elder Colleges’, which offer a wide range of courses and educational programs for older adults. A subset of the Elder Colleges were affiliated with a college or university, which provided technological resources such as online registration and scheduling systems, and support for instructors and students to navigate virtual learning platforms. Such supports were invaluable in promoting a smooth transition to online programming.

#### Intrinsic qualities of program design

Some participants described a stronger transition to online and hybrid activities due to intrinsic qualities of their existing programs. While multiple modes of communication to connect with members and deliver programming were occasionally utilized before the pandemic, the reliance on these methods over this period was considerably higher. Phone trees, messaging apps, email announcements, and physical mailing allowed for broad advertisement of their programs. Organizations also reported using both video-conferencing platforms and phone-based conferencing systems for program delivery. Organizations that used both modalities for activities reported that the modalities had different strengths and weaknesses and tended to attract different audiences. Video required access to IT devices with cameras and more technical knowledge and support, but enabled activities with a visual component, such as crafting or cooking classes. Telephone activities were simpler to set up for some older adults and provided a balance of privacy and intimacy that many program users appreciated.

Another important resource was an existing intergenerational focus within the organization. Established relationships with young people were re-purposed for weekly check-in phone calls and interest-based conversations by Zoom, telephone, or messaging app. Some of these connections also matched youth and seniors who spoke languages other than English. One organization reported a child-led cooking program on Zoom that had proven very popular with older adults. Some organizations also enlisted high school or university students to provide IT support. This approach garnered mixed reviews, one participant noting that young people tended to have difficulty understanding older adults’ needs, and another suggesting that young people who were not family members were well suited to this role.

Other program characteristics reported to facilitate uptake of virtual activities included having a wide variety of activities, keeping a regular schedule, and being proactive in contacting members about participation. Interviewed community organizations reported offering a huge range of social and educational programming, including games, arts and crafts, themed conversation circles, courses, book clubs, movie or music nights, sing-alongs, guided meditation, exercise (e.g., chair yoga), and guest speakers on topics of interest to older adults. Some activities were gendered (e.g., a men’s discussion group) and three organizations reported their activities had garnered more interest from women than men. The latter organizations were concerned that this imbalanced recruitment suggested males might be vulnerable to isolation. However, one organization described greater participation in Zoom sessions by men than women and was actively searching for ways to increase women’s participation. Moreover, several organizations mentioned that they needed a sufficient number of participants, typically 4–6 minimum, to make an activity sustainable in terms of invested resources such as staff and volunteer time.

#### Physical resources for virtual activities

Although virtual social interaction was the focus of our study, we found that virtual activities frequently relied on material and physical resources. Participants described that external grants were an important form of financial support to address inequities related to older adults’ lack of internet-connected IT devices. However, the application, competition, and reporting requirements associated with external funding were repeatedly described as a significant drain on the resources of smaller organizations, and the comparatively short time scales associated with many grants made long term planning more difficult. Community organizations described donations of IT resources such as smartphones, tablets, and Wi-Fi equipment by businesses in the community as valuable for supporting older adults’ use of IT for social purposes. Interestingly, home computers were not mentioned in any descriptions of received donations and were not prominent in any of the interviews. A few participants commented that computers in older adults’ homes were often obsolete and not useful for joining virtual activities.

Another affordance for virtual activities such as crafting, arts, or cooking on Zoom was scheduled drop-offs of physical goods. Volunteers delivered materials for specific arts programs such as cedar weaving or painting. Likewise, cooking sessions were supported by delivery of fresh ingredients. Despite significant logistical factors, community organizations reported that these programs had a significant payoff for member satisfaction and enjoyment.

#### Facilitators of technology use

Participants identified many facilitators related to IT. The most salient factors described by participants was access to specialized IT expertise within the organization. This included informal knowledge among leadership team or membership, a volunteer, or less frequently, a dedicated IT staff member. One all-ages community organization notably re-purposed their crew of youth lifeguards who possessed technological skills as phone support for seniors. Volunteers’ willingness to assist activity facilitators with setting up their sessions and helping members join was regarded as more important than specific IT expertise. At other times, volunteers provided one-to-one training and support for members as they familiarized themselves with new IT devices and platforms.

Community organizations reported supporting staff and volunteer activity facilitators by offering training in virtual program facilitation. In addition to learning how to navigate new IT platforms, training included a general introduction to best practices for delivering virtual sessions and how to manage interactions with program users during different types of activities (e.g., a conversation group vs. a lecture with Q&A). IT training for both facilitators and program users was greatly enhanced by finding or developing resources for common IT apps, platforms, and processes. In some cases, existing programs on using IT devices were also repurposed to support online activities. According to a couple community organizations, efforts to socialize members into virtual activities benefitted from a technological needs assessment to gauge ability, willingness, and interest to participate.

### III. Meeting the Challenge

Our final theme describes how community organizations responded to the need to rapidly and completely move to virtual programming due to the pandemic. Their insights have implications for future programming. First, community organizations reported increased participation by underserved members in their local communities, which we describe as increased ‘depth’ of reach. Second, community organizations noted new connections with members and other organizations across an expanded geographic range, which we name increased ‘breadth’ of reach. A handful of organizations were confident in the advantages of virtual activities. We note that these changes iteratively informed community organizations’ decisions about virtual and hybrid activities.

#### Increased ‘Depth’ of reach

A significant number of community organizations described how the essential transition to virtual programming had enabled them to reach new members within their local community. In some cases, these older adults were homebound due to physical factors including reduced mobility, allergies, or compromised immune systems. In other instances, prospective members had faced transportation barriers related to public transportation, driving long distances, and parking. Community organizations reported that older adults who lived in residential care facilities reportedly found themselves newly isolated when in-person recreation activities at their facility were restricted, or if their facility offered limited/no activities for people with linguistically and culturally diverse needs (e.g., a resident who primarily spoke Japanese in a facility where programming was offered only in English). Many of these previously unreachable older adults, including those who live in residential care facilities, found themselves, with the arrival of virtual programming, able to connect with peers in new and rewarding ways through community organization programming. Organizations lauded this new reach and reported considering keeping such approaches post-pandemic.

#### Increased ‘Breadth’ of reach

Almost as many organizations discussed how the online transition had enabled them to attract new participation from outside their previous geographic catchment area. This allowed for collaborations with groups like art museums, musical performances, and individual facilitators of courses, from other areas. Virtual activities also drew participants from distant locations; for example, an Indigenous program user took part in online drum circles from their location in Mexico, and program users who previously only participated in summer activities in B.C. before relocating for the Fall and Winter. Whether or not these members continued their patterns of travel during the pandemic, the ability to take part in programs remotely allowed many program users to participate during new times and in new ways. This new reach was appealing for community groups.

**Fig. 1:**
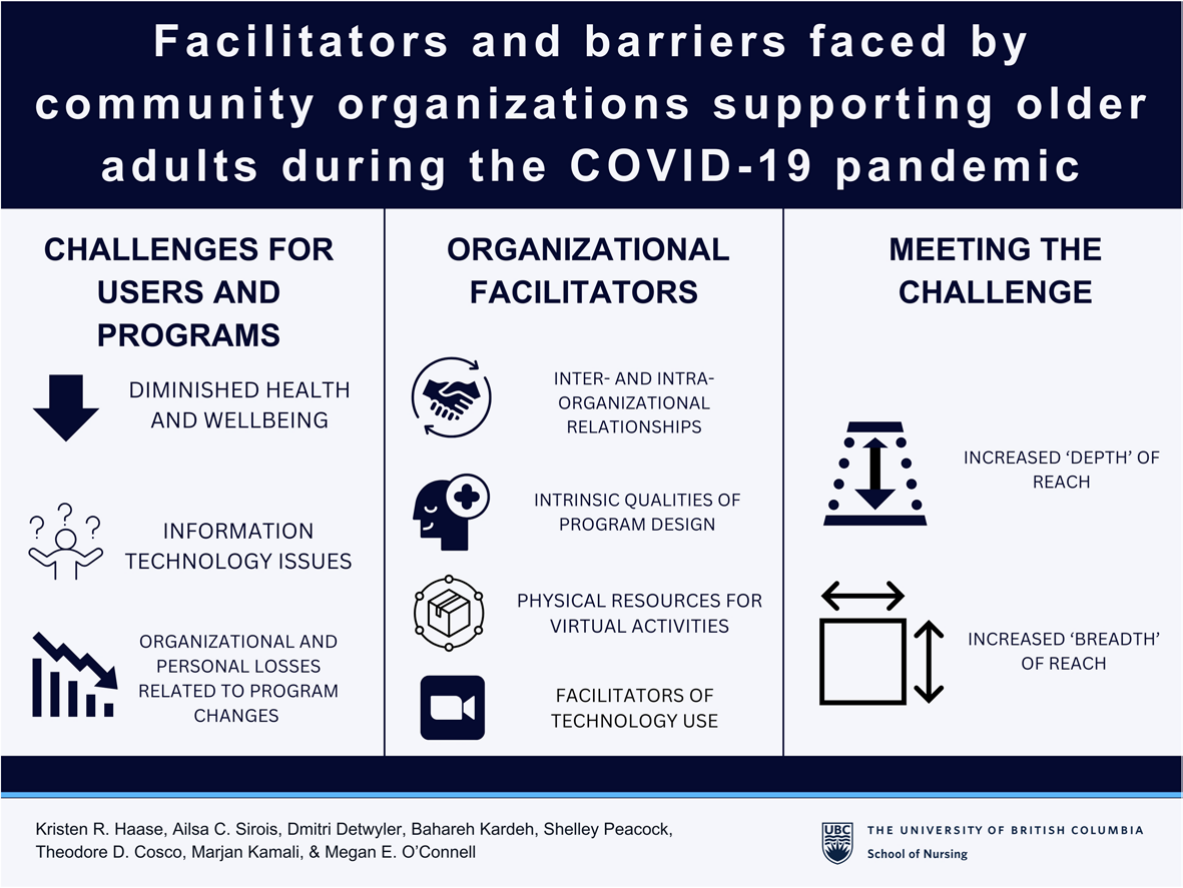
Visual summary of themes

Themes are summarized in Fig. 1.

## Discussion

Through this study we interviewed 26 individuals representing older adult-serving community organizations to understand their experiences of adapting their services during the COVID-19 pandemic. We included organizations from across B.C. serving older adults in rural, remote, and urban communities. Our most notable finding is the influence of resources as facilitators and barriers at both the organizational and individual level, commonly related to human resources and capacity to receive and provide IT support. Organizations with capacity to re-purpose staff (i.e. lifeguards with technical skills) were able to pivot their services to support the technological needs of older adults in their communities, whereas organizations that relied largely on volunteers struggled to adequately support their members. Despite the challenges, organizations lauded the benefits of increased reach in their membership activities facilitated by increased use of IT.

Findings related to the capacity of organizations to meet older adults’ needs have been reported elsewhere [34]. Somerville, Coyle and Mutchler [34] surveyed councils on ageing and senior centers in Massachusetts to understand adaptations made during the pandemic. Most of these organizations reported being very well prepared or somewhat prepared: most were still functional, but programs were limited to essential services, prioritizing nutritional and social needs. Results showed centers offering more programs were more likely to use both digital and traditional means than just either. This study also found challenges with IT, loss of staff, and adjusting to working remotely, which align with the findings in our study.

An important finding from this study relates to the value of community organizations that worked collaboratively to overcome pandemic-related barriers to increase their reach. Community organizations in BC found strength in numbers as they sought to address the needs of their combined members during the pandemic. Similarly, a recent study of the National Association for Area Agencies on Aging in the USA [35] showed that the majority of responding centers were serving more clients after the pandemic and with increased demand for aging services. This was due to transitions in ways of delivering services and the need to build new partnerships with community organizations such as healthcare entities. These partnerships helped mobilize services for the growing demands before additional funding was available.

Along with service delivery, community organizations can play an important role in distribution/dissemination of information in a time like the COVID-19 pandemic due to the established trust with their members. Weinberger-Litman et al. [36] studied a Jewish community who was the first community to be quarantined in the USA. The authors found that participants had greater trust in community organizations (religious institutions) compared to governmental and media sources of COVID-19 related information. This demonstrates the importance of close collaboration of health agencies with such communities to facilitate and ensure relay of reliable information. This is a salient finding as we combat the misinformation that accompanied the pandemic.

There were mixed findings about whether gender-specific programs were of more interest to men or women- however we note that gender differences for supports and coping existed before the pandemic [37–39]. One group described challenges engaging men in virtual activities which is consistent with prior research finding that men were underrepresented in community center use [39], whereas another organization reported the opposite. Although we did not find a gender analysis regarding transitions to online services published elsewhere, we note that Campos-Castillo [40] found that men are usually more likely than women to search for information but are less likely than women to share information. While our research is not conclusive related to the gendered implications of the pivot to online supports, it does warrant future consideration.

### Limitations

This analysis is subject to several limitations. We identified a large number of older adult-focused groups across the province, and of the groups contacted, a relatively low proportion had staff willing to be interviewed. Some declined to participate, citing limited resources and an overwhelming number of research-related requests. Others did not respond at all. As described in the methods section, interviews were not recorded. While not without limitations, this approach has been appraised as a suitable method for experienced qualitative researchers when conducting thematic analysis [29, 30]. We found this approach to be effective because participants were able to confirm the accuracy of the notes, which also became a resource for them. In an email communication, one participant noted the value of this systematic overview of their organization’s activities as a reference. We did not collect data on the size of the organizations, but most had few staff or were run entirely by volunteers, who may be older adults themselves. Of note, we were contacting the organizations between December 2020 and May 2021, a period of elevated COVID-19 case counts across B.C. and before and in the first days of the province’s vaccine rollout. We also recognize that our findings are a description of the experiences of local organizations with unique services and clients; the goal of our study was not generalizability. Further research in how community organizations supported older adults with IT during the COVID-19 pandemic can help inform policy and practice regarding older adults’ care and wellbeing beyond the pandemic.

## Conclusions

This study is important because it describes how community organizations leveraged available resources to maintain social interaction for older adults when physical distancing was required during the pandemic. The reliance on IT-based remote methods allowed community organizations to increase the depth of engagement with older adults, which facilitated engagement with linguistically and culturally diverse groups. Virtual methods also reduced travel burden and facilitated community organizations’ breadth of engagement. Nevertheless, this study documents significant barriers to IT access for older adults and community-based groups that serve them, which has implications for social interaction beyond the pandemic and for engagement in remote healthcare that continues to rely heavily on IT. Government investment in IT is needed – not only for community organizations, but also for older adults who may face sociopolitical barriers. Lack of access to or comfort with IT made a subgroup of older adults vulnerable to isolation during the pandemic, and underscores how IT use for older adults should be a target for policy and social services.

## Supplementary Information

Supplementary Material 1

## Data Availability

The datasets generated and analyzed during the current study are available from the corresponding author on reasonable request.

## Abbreviation

IT: Information technology
