# Supplementary material for: Facilitators and barriers faced by community organizations supporting older adults during the COVID-19 pandemic
Source: BMC Geriatr. 2025 Mar 28;25:204. doi: 10.1186/s12877-025-05816-w (PMC11951528; doi:10.1186/s12877-025-05816-w)
Supplement: Supplementary file 1 — Supplementary Material 1 [file 12877_2025_5816_MOESM1_ESM.docx]

**Supplement: Sample Interview Questions**

1. Tell me a little bit about your organization, including the purpose and user population?
2. Can you describe any challenges your program users have described as a result of COVID-19?
3. How have the programs or services delivered by your organization shifted over the course of the pandemic? What kinds of changes have you seen with your clients?
4. Can you describe the virtual socialization programs you are offering?
5. What has been the reception to the virtual programs?
   1. Are they well received ?
   2. What successes have you seen?
   3. What are some of the more significant gaps?
6. What kind of training and support do you offer, both for your staff working with older adults, and for your clients as you have moved to the virtual format?
7. Are you aware of **any other organizations** working in this space to provide virtual socialization opportunities for older adults that we should talk to?
